# Effects of Wolf Spiders’ Captive Environment on Their Locomotor and Exploratory Behaviours

**DOI:** 10.3390/insects13020135

**Published:** 2022-01-27

**Authors:** Marie Trabalon

**Affiliations:** EthoS-UMR 6552, CNRS, Université de Rennes 1, F-35000 Rennes, France; marie.trabalon@univ-rennes1.fr; Tel.: +33-223-237-101

**Keywords:** activity, experience, natural and artificial environments, male and female adult spiders

## Abstract

**Simple Summary:**

Since the 1960s, abuses of domestic and of wild animals that have been tamed or are held in captivity have been legally prohibited and laws ensure their well-being. Many scientific investigations carried out in this context recommend ways to adapt farming and thus to avoid physical and/or psychological suffering. Evaluations of animals’ welfare in captivity entail the need to understand in detail the fundamental behaviours of the focus species and to know the degree of their variation to be able to establish objective bases that can ensure breeding conditions that respect the animals’ welfare. Current laws do not apply to invertebrate animals (such as insects or spiders) and consideration of the welfare of these animals in captivity is neglected. Here, I compared the behaviour of wild adult spiders just after collection and that of adult spiders hatched and bred in the laboratory. My results show that captivity induced rapid changes of wild spiders’ behaviour once in captivity. Therefore, it is important to establish the best breeding conditions for the needs of both invertebrate and vertebrate animals in order to promote their well-being.

**Abstract:**

Here I detail the effects of the abiotic/captive environment of an adult wandering spider, *Pardosa saltans* (*Lycosidae*) on its behaviour. These studies focused on spiders collected as adults in their natural environment and spiders developed in the laboratory under controlled conditions. Wild-caught spiders were tested either immediately after capture or after being housed for 15 days post-collection. Laboratory reared spiders were kept in different environments: small or large space combined with the presence or absence of litter. Two tests evaluated by sex show the influence of these rearing conditions: an open-field test and a radial-arm maze test. The results show that wild caught spiders of both sexes tested immediately after capture weighed significantly less and were significantly more active than spiders housed in the laboratory for 15 days and spiders reared in the laboratory. Laboratory conditions induced a positive impact on body mass and negative impact on behaviour activities. The locomotor and exploratory activities of spiders of both sexes kept in container without substrate showed lower. My results suggest that the physical enrichment of the environment can reduce these negative effects for females, but not for males that seem to be more affected by being reared under controlled conditions.

## 1. Introduction

For a few decades now, consideration of animal welfare has been growing in scientific circles, the food industry, legislation, and even at private and individual levels. In this context have emerged many studies focusing on the impact of captivity and breeding conditions on the well-being and, more generally, on the behavioural characteristics of many vertebrates [1,2]. Thus, it has been general knowledge for some time now that the enrichment of an individual’s environment is an important factor that must be taken into consideration to ensure that an individual’s coping skills and physical health are good and that it develops a rich behavioural repertoire [3,4]. These reports show that the well-being of domestic animals as well as that of animals in captivity can be improved by enriching and complexifying their captive environment and by providing sufficient space. Levels of locomotor and exploratory activities are good indicators of an animal’s well-being. Thus, a way to estimate the level of well-being of animals in captivity is to compare their levels of locomotor and exploratory activities to those of animals in their natural environment. Studies of this type but focusing on invertebrates are rare. However, we know that, as for vertebrates, environmental constraints experienced during ontogeny can have important effects on the morphological and physiological development of an invertebrate, as well as on the establishment of its behavioural repertoire, as for example its locomotor and exploratory behaviours.

Locomotor and exploratory activities reveal the tendency of all animals to move about and inspect their environment, even when neither hunger, nor thirst, nor sexual appetite compels them to do so. These behaviours allow an individual to gather a certain amount of information concerning their environment and are adapted to its living environment. In the event of a change in its environment during its lifetime an individual has to adapt its behaviour to the novel characteristics of its environment, an acclimatization that may involve a change in its behaviour. According to the literature, locomotor and exploratory activities are subject to environmental constraints (abiotic and biotic), genetic constraints (sexual dimorphism) and maturation constraints of an organism (physiological state). For example, a decrease in ambient temperature results in a decrease of the locomotor activities of most arthropods [5] and the presence of conspecifics in the environment induces an increase in their exploratory behaviour [6,7].

Sexual maturity causes a modification of the exploratory and locomotor activities of the majority of arthropod species and exploratory behaviours can then differ between adult males and females (spiders: [8]; myriapods and ground beetles: [9,10]; woodlouse: [11]). Generally, adult male arthropods move about more and this tendency can be explained by the fact that males, in addition to foraging, are looking for a sexual partner [12]. Conversely, females move about less, remaining almost in the same place to allow males to find them more easily [13].

Over the past years, a few authors have been interested in the impact of the rearing environment on different species of spiders [14]. Thus, some studies compare the locomotor and exploratory activities of individuals captured in their natural environment to that of individuals reared in an artificial environment. For example, *Phidippus audax* (*Salticidae*) spiders captured in the wild approach artificial prey faster and from a more distant position and are less static than individuals that had hatched and been reared in the laboratory [15,16,17]. Folz [18] showed that *Hogna helluo* (*Lycosidae*) spiders reared in smaller terraria were faster to search for and capture prey. Other observations showed that individuals that had developed in a physical environment enriched were more active than individuals maintained in a non-enriched environment [6,15,19,20].

My study aims to highlight differences between wandering spiders that developed in the laboratory and spiders that had developed in their natural environment. I hypothesised that the locomotor and exploratory behaviours of spiders reared in the laboratory would differ from those of spiders that had developed in their natural environment because of restricted living conditions in the laboratory. In addition, I evaluated the effects of different types of rearing conditions on the behaviour of female and male adults to evidence factors that could improve their living conditions. In this context, I questioned whether the size of their terraria would modify the spiders’ behaviour in relation to the volume in which they had developed. I assumed that spiders reared in large terraria would be more active than those reared in small terraria. Second, I questioned whether the presence of litter in a terrarium would induce any behavioural changes. I assumed that a complex environment such as undergrowth litter would induce individuals to move about and explore their environment more. Third, I evaluated how long in captivity after being collected the behaviour of wild caught adult spiders would be modified. The subjects of most laboratory studies on reproduction behaviours (sexual and maternal) are individuals that have been kept in the laboratory for a long time or under maintenance conditions that affect individuals’ behaviour. I assumed that 15 days of captivity would not lead to any behavioural modification. My model spider species is the free-moving wolf spider, *Pardosa saltans* (*Lycosidae*).

## 2. Materials and Methods

### 2.1. Ethics Statement

By using an invertebrate species and caring for it while using the accepted ethical standards in the laboratory, my research conforms to the legal requirements and guidelines established for the treatment of animals in research. The species used for these experiments is neither endangered nor protected.

### 2.2. Spider Collection and Rearing

Members of the species *Pardosa saltans* can be found in forests, woodlands and thickets, and sometimes near grasslands and hedges. Their distribution appears to be largely restricted to old and ancient woodland sites, where they can become numerous, running over the ground in open clearings as well as amongst litter in the shade of a wood. The breeding season of *P. saltans* extends from March to September in France [21]. During the day, *P. saltans* spiders move ceaselessly over the substrate in search of food, a sexual partner or a sunny area to warm up. These spiders hide under the litter at the end of the day and until the morning to protect themselves from high temperature variations and predators. It is for these various reasons that *Pardosa saltans* is an ideal model to study locomotor and exploratory behaviour.

Two experimental groups were used: spiders that had emerged and developed in their natural environment (natural spider group) and spiders that had emerged and been reared in the laboratory (laboratory spider group).

**Figure 1:**
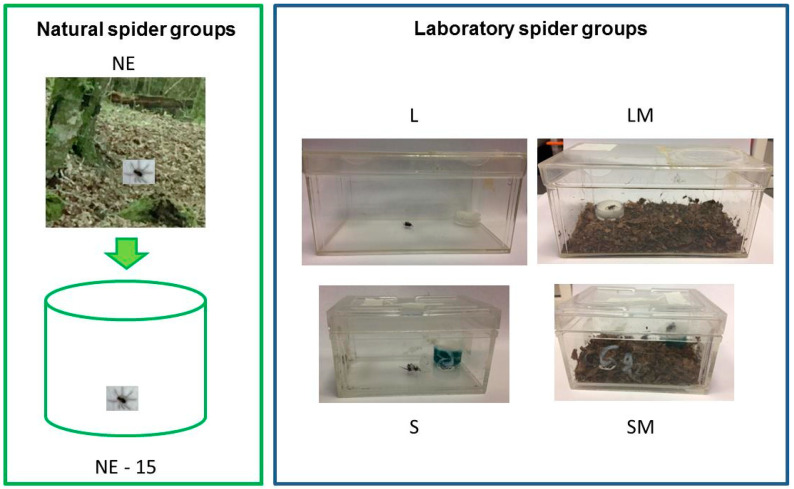
Rearing conditions of adult spiders: developed in natural environment (NE group); wild caught spiders housed in the laboratory for 15 days (NE-15 group); juveniles spiders developed in the laboratory in large terraria without matter on the base until adult (L group); developed in large terraria with matter on the base until adult (LM group); developed in small terraria without matter on the base until adult (S group); developed in small terraria with matter on the base until adult (SM group).

*Natural spider group:* The subjects were female and male adults captured in a private forest near Guichen (France; 47°58′03″ N, 1°47′43″ W) in April-May 2021. Spiders were randomly assigned to two experimental groups (Figure 1). Adults of first group were weighed (Sartorius electronic balance, ±0.01 g; Palaiseau, France) then tested between 2 and 3 h after collection and returned to their natural environment at the end of the behavioural tests (NE group). Adults of second group were weighed just after collection and then kept individually during 15 days in circular terrarium (15 cm in diameter, 5 cm high) with water and without soil, under the same temperature as outdoors during these days (22 ± 2 °C during day and 16 ± 3 °C during night; and hygrometry (37 ± 5% relative humidity) and natural photoperiodic cycle (NE-15 group). These NE-15 spiders were fed every five days ad libitum with juvenile crickets (*Acheta domestica*) alternating with adult flies (*Delia radicum*). After 15 days in captivity, spiders were weighed, behavioural tested and then returned to their natural environment.

*Laboratory spiders groups*: Juveniles spiders (*n* = 120) emerged from six cocoons in the laboratory during September 2020 and were reared in the laboratory until they were adult (April–May 2021). The juvenile spiders (7–8 days after emergence), after they had caught their first prey, where kept individually either in large (L × l: 17 × 9 cm, 8 cm in high) or small transparent terraria (L × l: 9 × 6 cm, 5 cm in high). Both large (L) and small (S) terraria were divided into two subgroups: either with 1 cm litter (mixture of earth and leaves from their native forest) on the base, or without any matter on the base (Figure 1). These spiders were kept for seven months under natural temperature (20 ± 4 °C during day and 10 ± 3 °C during night), hygrometry (37 ± 5% relative humidity) and natural photoperiodic cycle. They were fed every six days ad libitum, with juvenile crickets (*Acheta domestica* and *Nemobius sylvestris*) alternating with adult flies (*Delia radicum*). After seven months of development, all spiders were checked every day to record their adult moult and tested between 2 and 3 weeks after their adult moult.

### 2.3. Locomotor and Behaviour Activities of the Natural Spider Group in the Laboratory

The activities of the natural spiders (*n* = 25/sex) were video recorded in their terrarium the day they were collected just after they had been placed in their terrarium (day 0 in captivity) then 7 (7 days in captivity) and 14 days after collection (14 days in captivity), by a camera (video tracking). All observations were recorded between 14.00 and 17.00 h in natural light with the SMART-MA software program (Smart Panlab, Bioseb, France). The software program calculated directly locomotor activity (time of mobility, trajectories) expressed in surface explored (cm/min) (Table 1). Movements without changing place were recorded directly by the experimenter using a keyboard event recorder integrated in SMART-Ma system (key were designated as behaviour events). Six spontaneous behavioural events without moving were observed and recorded (Table 1). The program allows recording duration of events versus frequency of events. Timing of events is accurate to 0.1 s. During captivity in a terrarium, “abdominal” vibrations were manifested only by females and “drumming” only by males.

### 2.4. Locomotor and Exploratory Activity in a Novel Environment

Six experimental groups (*n* = 25 males and 25 females in each experimental group; Figure 1) were observed in two novel environments and each spider was only tested once: -NE group: female and male adults that had developed in their natural environment tested between 2 and 3 h after capture; -NE-15 group: female and male adults that had developed in their natural environment tested after 15 days in captivity; -L group: female and male adults that had developed in the laboratory in large terraria without any matter on the base; -LM group: female and male adults that had developed in the laboratory in large terraria with matter on the base; -S group: female and male adults that had developed in the laboratory into small terraria without any matter on the base; -SM group: female and male adults that had developed in the laboratory in small terraria with matter on the base. All subjects were observed at the same time of day (between 14.00 and 17.00 h), at 20 ± 1 °C with 40 ± 2% relative humidity in natural light (90 lx in each glass arena, measured by a Spengler Luxmeter before each behavioural test, Securimed, Cappelle-La-Grande, France).

**Figure 2:**
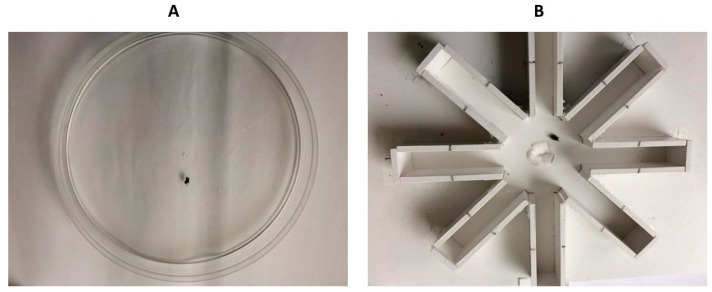
The open-field glass arena (**A**) and the eight radial-arm maze (**B**) used successively during behavioural tests for wild caught spiders immediately after capture (NE) or after 15 days in captivity (NE-15) and for laboratory reared spiders (L, LM, S and SM groups).

Each spider was observed successively in an open-field glass arena (15 cm in diameter, 5 cm high covered with a glass plate) and in a radial-arm maze (Figure 2). The maze was an array of eight arms in opaque white PVC (L × l: 10 × 2.5 cm and 3 cm high) radiating from a central starting area (6 cm in diameter) and covered with a transparent glass plate (L × l: 22 × 24 cm). Two arenas and two mazes were used simultaneously during a test so we could pair subjects in relation to sex and experimental environment, combining all possibilities (L/LM, L/S, L/SM, L/NE, L/NE-15, LM/S, LM/SM, LM/NE, LM/NE-15, S/SM, S/NE, S/NE-15, SM/NE, SM//NE-15, NE/NE-15). This methodological precaution was taken to counteract effects of possible changes in temperature and humidity on spiders’ behaviour. To eliminate biases due to the positions of the arenas, I alternated the experimental groups between sides and between trials. The arenas were washed with acetone-water (5%) then ethanol (70%) between trials to eliminate any possible intraspecific cues (odour and dragline cues left by the previous spider) that could influence a spider’s activity. They were reused 1 h after evaporation of washing solvents.

Spiders were observed individually in the arena and their behaviour and locomotor activities were recorded and analysed applying the method followed by Ruhland et al. [21]. The coordinates of spiders in the novel environment (arena and maze) were recorded every 12 frames using a Canon HD (HG20) camera and an automated video-based, digital-data collection system (Swiss-Track software 4.0.0 with the nearest neighbour tracking method).

After being weighed, a spider was placed in the centre of the arena under a bell (1.5 cm in diameters) 1 min prior to the test. The bell was removed, and the subject was allowed at least 2 min to acclimate to the arena prior to data collection. After the acclimation period, the spider’s activity (locomotors activity and spontaneous activity without changing place; Table 1) was monitored for 10 min. The software program calculated a spider’s locomotor activity directly (expressed in surface explored, cm/min). Other movements in the arena were recorded directly by the experimenter as indicated above (Table 1).

At the end of arena test, the spider was rapidly transferred into a vial and immediately placed in the central box of the radial arm maze under a bell (1.5 cm diameters) for evaluation of the exploratory activity. The bell was removed, and the spider was allowed at least 2 min to acclimate to the arena prior to data collection. After the acclimation period, the spider’s exploratory activity in the maze was monitored during 10 min (Table 1). The software program calculated exploratory activity (time of mobility, number of arms visited) expressed in distance covered (cm/min) of each spider directly. When a distance covered was less than 3 mm/second it was not taken into account.

### 2.5. Statistical Analyses

Statistical analyses were performed using STATISTICA 6.0 for WINDOWS (Statsoft Inc., Tulsa, OK, USA). A generalised linear mixed test (GLMM, negative binomial distribution) was conducted with data of body mass, the surface explored and distance covered after transformation of data in log to normalize the data. Frequencies of spontaneous behaviours were divided by 100 and then converted to √arcsine proportions to normalize the data before a generalised linear mixed test was conducted. When differences among means were significant at the *p* < 0.05 levels, an a posteriori Wald test was used to establish inter-group comparisons. Means are given ±95% confidence intervals (CI).

## 3. Results

### 3.1. Body Mass

**Figure 3:**
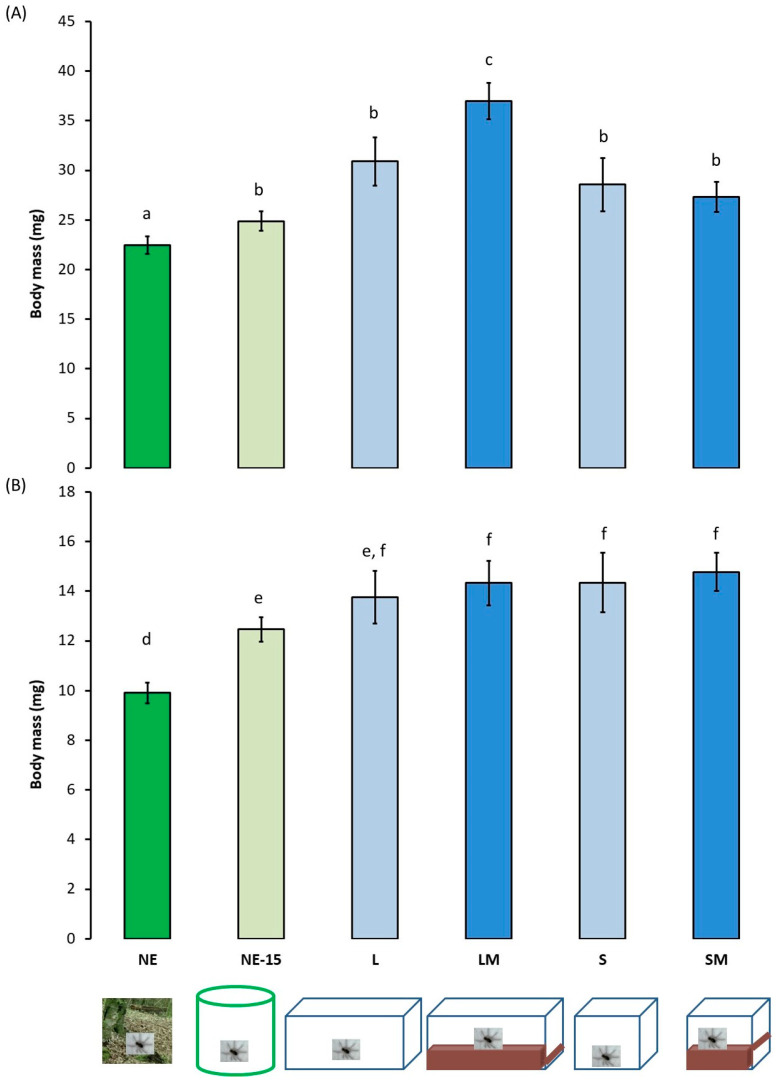
Body mass ±95% confidence intervals (in mg) of adult female (**A**) and male (**B**) *Pardosa saltans* in relation to the environment where they developed: in their natural environment and tested between 2 and 3 h after capture (NE); in their natural environment and tested after 15 days in captivity (NE-15); in the laboratory in large terraria without matter on the base (L); in the laboratory in large terraria with matter on the base (LM); in the laboratory in small terraria without matter on the base (S); in the laboratory in small terraria with matter on the base (SM). Data were compared using generalised linear mixed mod (GLMM, negative binomial distribution). Different letters indicate a significant difference at *p* < 0.050, post hoc Wald test.

Body mass (Figure 3) differed significantly between spider groups and between sexes (environment effect: 4.095, df = 5, *p* < 0.001; sex effect: 20.886, df = 1, *p* < 0.001; environment*sex: 0.349, df = 5, *p* = 0.083). Body mass of NE females was significantly less than that of NE-15 females and laboratory reared females (Figure 3A). Body mass of LM females was significantly greater than that of all the other females. All males were significantly lighter than females and body mass of NE males was significantly less than that of NE-15 males and of males of all the laboratory-reared groups (Figure 3B).

### 3.2. Behaviour and Locomotor Activity of NE Spiders

**Figure 4:**
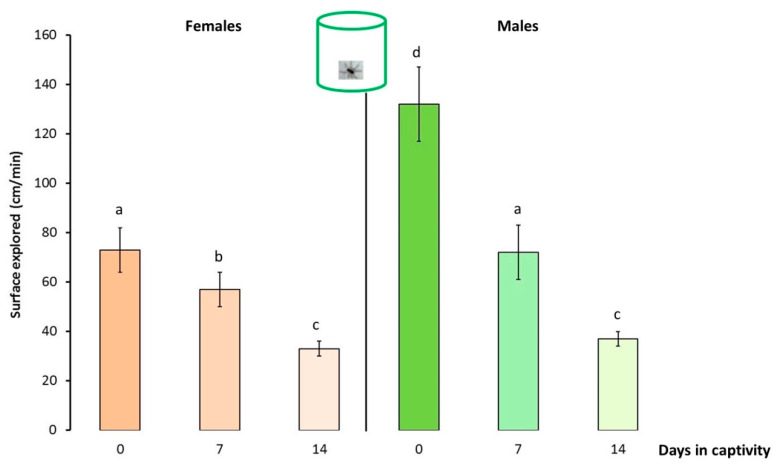
Locomotor activity ±95% confidence intervals (expressed in surface explored, cm/min) of adult female and male *Pardosa saltans* in terraria after capture in their natural environment (0 day in captivity), seven days after capture (7 days in captivity) and 14 days after capture (14 days in captivity). Data were compared using generalised linear mixed mod (GLMM, negative binomial distribution). Different letters indicate a significant difference (*p* < 0.050, post hoc Wald test).

*Pardosa saltans* moved about actively in their terrarium (Figure 4) and locomotor activity of the natural spider group varied significantly with time and between sexes (day effect: χ = 128.105, df = 2, *p* < 0.001; sex effect: χ = 4.289, df = 1, *p* = 0.038; day*sex: χ = 12.558, df = 2, *p* = 0.002). Females and males after capture (0 day in captivity) mover significantly more than did the other. After 7 then 14 days in captivity, the locomotor activity of all the natural spiders decreased significantly and the locomotor activity levels of females and males became similar after 14 days in captivity.

**Figure 5:**
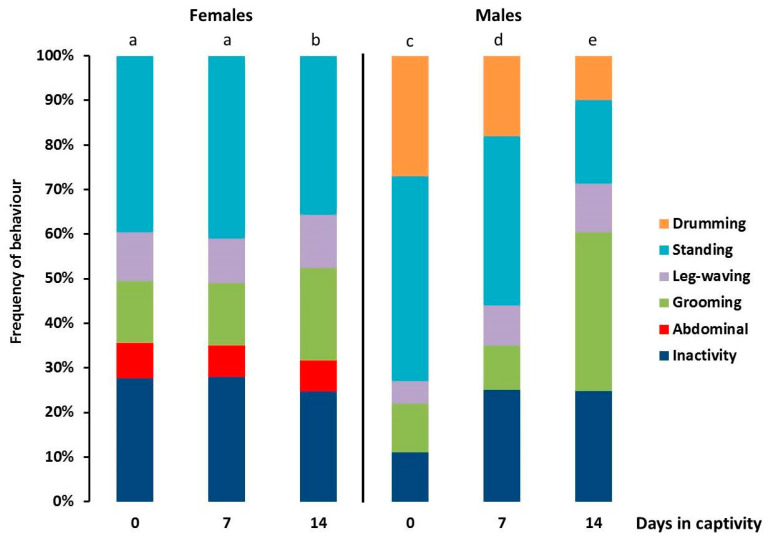
Spontaneous behavioural activities without changing position of adult female and male *Pardosa saltans* (in frequencies) in terraria after capture in their natural environment (0 day in captivity), seven days after capture (7 days in captivity) and 14 days after capture (14 days in captivity). Frequencies of behavioural events were compared using generalised linear mixed mod (GLMM, negative binomial distribution). Different letters indicate a significant difference (*p* < 0.050, post hoc Wald test).

The frequency of different behaviours (Figure 5) varied significantly between sexes and with time (sex effect: χ = 6.61 to 7.47, df = 1, *p* = 0.010 to 0.006; day effect: χ = 8.82 to 1057.42, df = 2, *p* = 0.012 to < 0.001; day*sex: χ = 17.97 to 273.42, df = 2, *p* < 0.001). Males were significantly more active than females. Females and males significantly increased “grooming” and decreased “standing” after 14 days in captivity. As time since collection increased males became more inactive and increased “grooming” and “leg-waving” activities.

### 3.3. Behaviour in a Novel Environment (Open-Field Arena and Arm-Maze)

**Figure 6:**
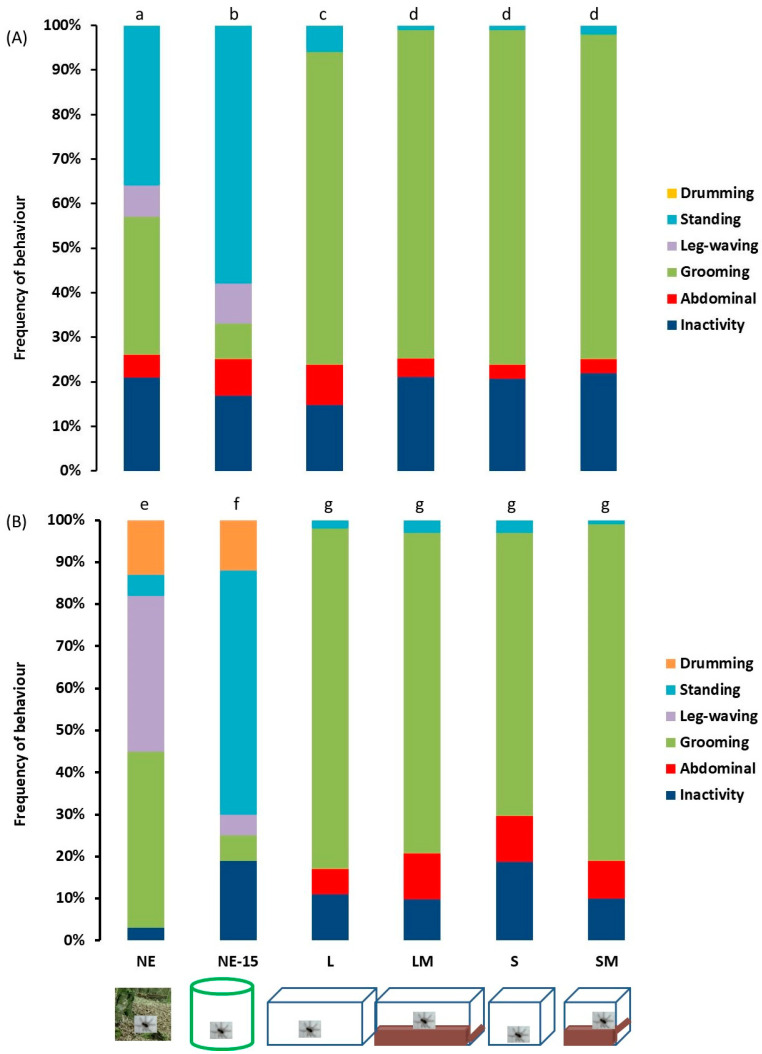
Spontaneous behavioural activities without changing position of adult female (**A**) and male (**B**) *Pardosa saltans* (in frequencies) in the open-field arena in relation to the environment where they developed: (NE) in their natural environment and tested 2–3 h after capture; (NE-15) in their natural environment and tested after 15 days in captivity; (L) in the laboratory in large terraria without matter on the base; (LM) in the laboratory in large terraria with matter on the base; (S) in the laboratory in small terraria without matter on the base; (SM) in the laboratory in small terraria with matter on the base. Frequencies of behaviours were compared using generalised linear mixed mod (GLMM, negative binomial distribution). Different letters indicate a significant difference (*p* < 0.050, post hoc Wald test).

Levels of behavioural activities in a novel environment (open-field arena) varied significantly between spider groups (Figure 6) and between sexes (environment effect: χ = 46.21 to 1924.87, df = 5, *p* < 0.001; sex effect: χ = 20.50 to 162.82, df = 1, *p* < 0.001; environment*sex: χ = 14.25 to 1075.06, df = 5, *p* = 0.010 to < 0.001). Only the natural spider group manifested “leg-waving” and only natural males manifested “drumming” activity. The natural females presented significantly more “standing” and less “grooming” than did the females of all the laboratory groups. The natural males manifested significantly less “grooming” and “inactivity”, and more “standing” than the other males. Levels of spontaneous behavioural activity without changing position did not vary significantly between the laboratory female and male groups.

### 3.4. Locomotor and Exploratory Activities in a Novel Environment

**Figure 7:**
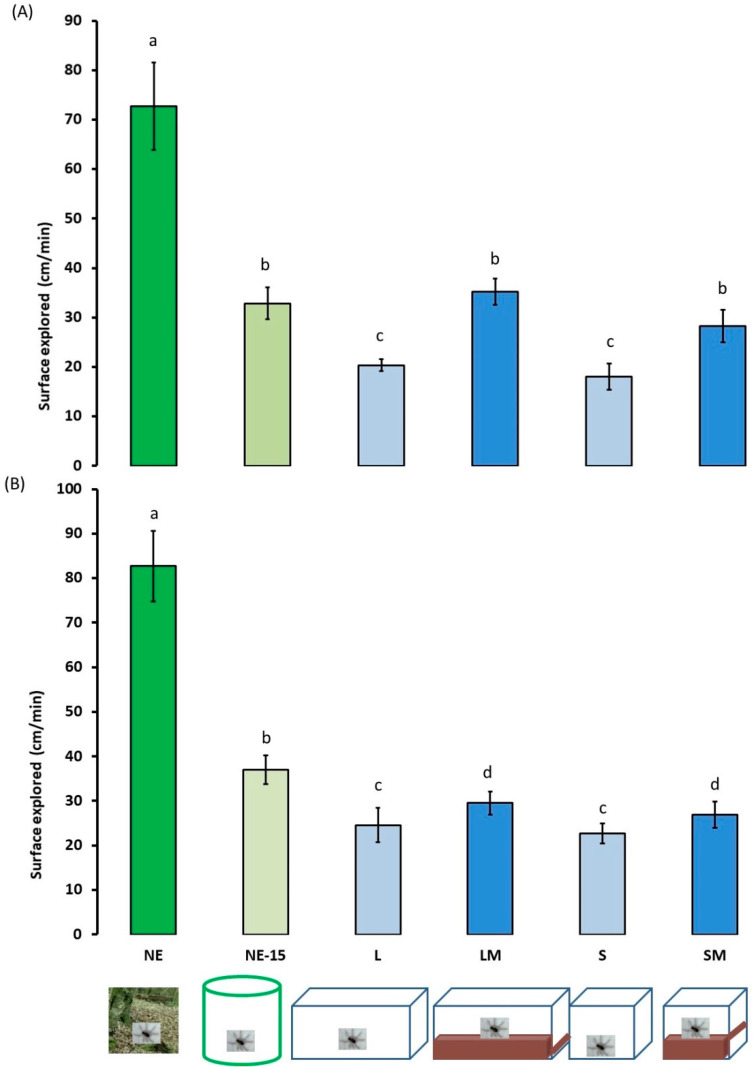
Locomotor activity ±95% confidence intervals (expressed in surfaces explored, cm/min) of adult female (**A**) and male (**B**) *Pardosa saltans* in a open-field arena in relation to the environment where they developed: (NE) in their natural environment and tested 2–3 h after capture; (NE-15) in their natural environment and tested after 15 days in captivity; (L) in the laboratory in large terraria without matter on the base; (LM) in the laboratory in large terraria with matter on the base; (S) in the laboratory in small terraria without matter on the base; (SM) in the laboratory in small terraria with matter on the base. Data were compared using generalised linear mixed mod (GLMM, negative binomial distribution). Different letters indicate a significant difference (*p* < 0.050, post hoc Wald test).

**Figure 8:**
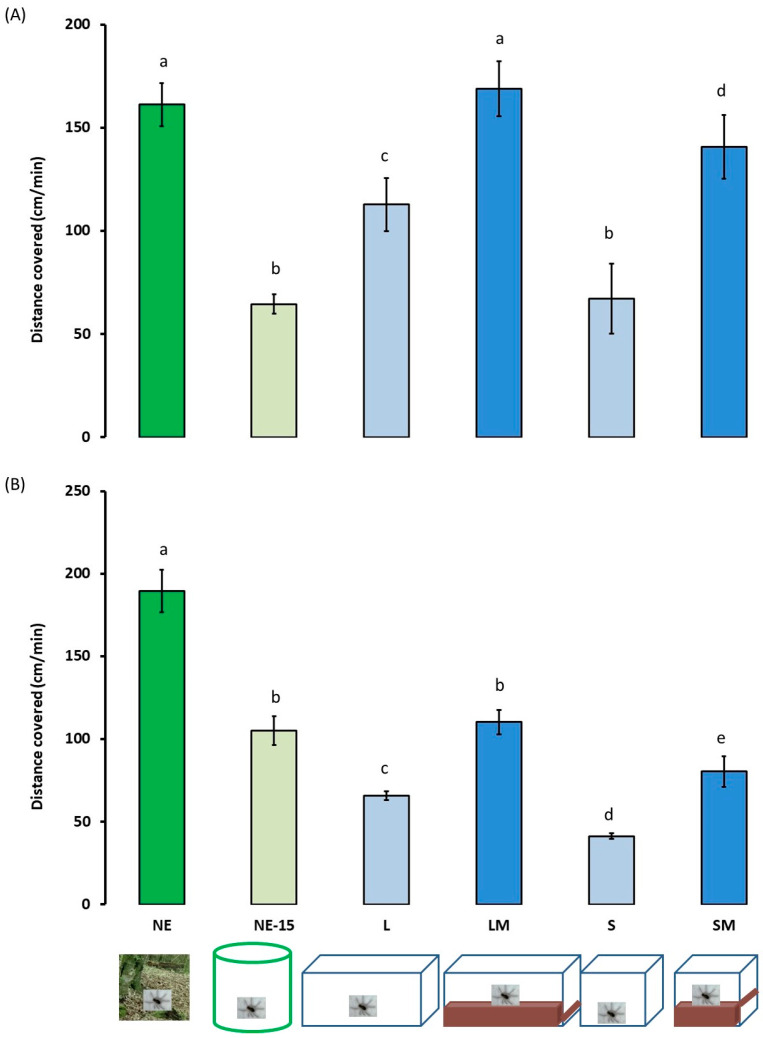
Exploratory activity ±95% confidence intervals (expressed in distances covered, cm/min) of adult female (**A**) and male (**B**) *Pardosa saltans* in a radial arm maze in relation to the environment where they developed: (NE) in their natural environment and tested 2–3 h after capture; (NE-15) in their natural environment and tested after 15 days in captivity; (L) in the laboratory in large terraria without matter on the base; (LM) in the laboratory in large terraria with matter on the base; (S) in the laboratory in small terraria without matter on the base; (SM) in the laboratory in small terraria with matter on the base. Data were compared using generalised linear mixed mod (GLMM, negative binomial distribution). Different letters indicate a significant difference (*p* < 0.050, post hoc Wald test).

All spiders walked preferentially close to the periphery of the open-field arena and radial arm maze. Surfaces and distances explored during locomotor activity in the open-field arena and the radial arm maze (Figure 7 and Figure 8) varied significantly between the experimental spiders groups (environment effect: χ = 13.473, df = 5, *p* < 0.001; sex effect: χ = 2.046, df = 1, *p* = 0.155; environment*sex: χ = 0.264, df = 5, *p* = 0.932 for surfaces explored; environment effect: χ = 13.325, df = 5, *p* < 0.001; sex effect: χ = 8.386 df = 1, *p* = 0.004; environment*sex: χ = 3.399, df = 5, *p* = 0.006 for distance covered). Thus, NE females and males moved about more: they explored larger surfaces and covered greater distances. After 15 days in captivity the surfaces explored by females and males did not differ significantly from the surfaces explored by LM and SM females. The surface explored and distances covered by the L and S spiders were significantly lower.

## 4. Discussion

In this study, I used a combination of behavioural assays to examine the effects of rearing conditions on the locomotor, exploratory, and spontaneous behavioural activity of the wolf spiders, *P. saltans*. My objective was to answer mainly three questions: does captivity, after only 15 days, affect the locomotor and exploratory behaviour of adult spiders developed in their natural environment? Does captivity affect female and male adults differently? Should wandering spiders be raised and maintained in large or small terraria, with or without litter? My results show that activity levels varied based on sex and treatment.

When introduced into a novel environment immediately after capture, my results show that adult *P. saltans* spiders developed in the wild exhibit high levels of locomotor and exploratory activity. Males are twice as active as females as previously observed in other studies with other invertebrate species [8,9,10,11,22]. Thus, males explore larger surfaces but contrary to our predictions they do not cover significantly longer distance than the females. During phases of immobility, spiders exhibit six spontaneous behaviours: “inactivity”, “abdominal vibration”, “grooming”, “leg-waving”, “standing” and “drumming”. “Grooming” of the legs and pedipalps are the most frequent activities observed. These activities can be explained by the fact that adult spiders possess many chemoreceptors (receptors for chemical signals) and mechanoreceptors (receptors for vibratory signals) on the tarsi of their legs and their pedipalps [23,24]. These sensory receptors are highly stressed when travelling in heterogeneous environments and individuals must regularly remove, by “grooming”, any artefacts that could impair the perception of communication signals. “Grooming” activities increase the reception of communication signals when an individual is foraging or sexually active, especially in a novel environment. In addition, males mainly manifest “leg-waving” and “drumming” activities, characteristically emitted by adult male spiders during sexual activities when searching for a sexual partner in their natural environment [8,16]. Females placed in a novel environment mainly perform “standing” against the walls of the experimental enclosures.

Captivity results in rapid behavioural acclimatization of spiders that had developed in their natural environment. Thus after 7 days of captivity, the locomotor and exploratory activities of both female and males spiders decreased significantly. After 15 days in captivity, these spiders moved significantly less in their terraria and the levels of locomotor activity of females and males during behavioural assays were comparable to those of individuals reared in the laboratory in small terraria without substrate. These results corroborate observations of a salticid spider *Phidippus autax* maintained under artificial conditions for 4 months [15]. This decrease was not correlated with a change in ambient temperature and/or humidity at the time of testing, unlike for other animals [25,26,27]. This decrease was also not correlated with a lack of energy as these female and male spiders had put on weight. Consequently, the decrease in activity shown by spiders from the natural environment is clearly linked to an effect of captivity.

Captivity also results in a change in the spontaneous behavioural activities exhibited by the spiders developed in their natural environment as observed for other animal species [2,19]. It is the males who are the most affected by captivity. Thus after only 7 days of captivity, the locomotor activity of males is comparable to that of females. This gradual decline is accompanied by a decrease of “standing” and “drumming” activities and, conversely, by an increase in “grooming” and “inactivity”. This change in behavioural activities of females is less visible, although they, like males, show a significant decrease in their locomotor activity after 14 days in captivity. Thus, a change of environment in adulthood leads *P. saltans* to gradually, significantly but quickly modify its locomotor and exploratory activities. This effect is perhaps linked to the sudden modification of their environment, which induces greater deprivation for individuals accustomed to moving freely over large surfaces and in a complex physical environment. Thus, my study shows that being kept in a terrarium induces a progressive and negative modification of the behavioural activities of *P. saltans* spiders, as observed for three species of tarantulas [28]. In their natural environment, *P. saltans* moves in different environments such as undergrowth and meadows. These spiders are therefore able to adapt quickly to a novel environment, which explains the rapid change in their behaviour the days following their collection.

With regard to effects of breeding on spiders, my results show that the size of the terraria did not appear to affect the spontaneous behaviour or the locomotor activity of adult spiders: female and male spiders reared in either small or large terraria exhibit the same spontaneous behaviours and the same levels of surface explored contrary to observations by other researchers on Salticid spiders [15,18,19]. However, the size of the terraria affects their exploratory activity: spiders, and more particularly males, reared in small terraria cover shorter distances when exploring a novel environment. These differences in activity are once again not linked to abiotic factors (temperature, humidity) or to the weight of the spiders. Indeed, the spiders bred in the laboratory and according to sex have comparable masses.

Conversely, the presence of litter in terraria promotes the levels of *P. saltans* spiders’ locomotor and exploration activities as for *Marpissa muscosa* spiders [6]. Thus, females and males reared in terraria with litter are more active, cover greater areas and distances than spiders reared in terraria without litter. These results show that the presence of substrate stimulates favourably the locomotor and exploratory activities of wandering spiders during their development as for vertebrates [29,30]. However, spiders that have developed in their natural environment are generally more active, move and explore more than those that have been reared in the laboratory even in the presence of litter. In addition, males reared in the laboratory, even in the presence of litter in their terrarium, show low levels of locomotor and exploratory activities comparable to those of females. My maintenance conditions in an artificial environment are too restrictive for the simple fact of adding a substrate or using a larger terrarium to be sufficient to compensate deprivations related to these breeding methods, in particular for males.

Furthermore, the spontaneous activities exhibited by the spiders reared in the laboratory during the behavioural tests show homogeneity between the different experimental groups. Neither the size nor the presence of litter influences the behavioural activity of the spiders that had developed in captivity for seven months. These female and male spiders exhibit “grooming” and “abdominal” vibrations when exploring a novel environment. The spiders reared in the laboratory show no “drumming” or “leg-waving” activities. These activities are used by adult spiders during the search for a sexual partner. These results suggest that these behavioural activities may emerge only as a consequence of being reared in a social environment, as observed for *Brachypelma smithi* tarantulas [19]. In addition, in their natural environment, spiders regularly encounter conspecifics. These encounters elicit the manifestation of behavioural characteristics of both their species and sex such as “leg-waving” and “drumming”. The spiders in the laboratory group had been reared in social isolation during the seven months their development lasts, that is, they had never been in contact with any conspecifics. This would explain why female and male spiders reared in the laboratory are less active and do not “leg-waving” or “drumming”. The absence of these behaviours indicates that these activities are linked to social learning that takes place during development, and that social isolation induces a reduction in behavioural complexity as observed for vertebrates [2,31,32,33,34]. Additionally, this socially deprived environment probably affects learning abilities and the central nervous system of *P. saltans* as observed for the jumping spider, *Marpissa muscosa* [35,36].

On the other hand, it is interesting to note that the body mass of *Pardosa saltans* differs according to sex and environment. Males weighed less than females, confirming previous studies of these spiders [37], and the spiders collected in the wild were lighter than those that have been raised for seven months in the laboratory in a controlled environment. After 15 days in captivity, rearing conditions affected positively their weight gain. Furthermore, males reared in the laboratory weighed less than laboratory reared females. However, under laboratory conditions, the males were fed ad libitum and maintained under relatively stable abiotic conditions during development (e.g., no rain, no frost). Under these conditions, one might think that these males would probably spend less energy to fight heat loss and to explore their environment in search of food compared to other males subjected to environmental constraints, and therefore they would be able to store more energetic stock in their organism. My results showed that this is not the case, so I hypothesised that inter-individual mass variations are influenced more by genetic factors than by biotic or abiotic environmental factors. The fact that my results concerning females differ from those for males suggests that regulation of weight gain differs in relation to sex. Indeed, the body weights of females varied according to their living environment. Females from the natural environment weighed less than females reared in the laboratory in a controlled environment and fed ad libitum. In addition, females reared in large terraria weighed more than females living in small terraria. *Pardosa saltans* females therefore exhibit morphological plasticity allowing individuals to adapt to the spatial characteristics of their captive environment when their diet is optimal. This result can be linked to observations of other animal species kept in controlled environments (e.g., fish in aquariums: [38]; lobsters: [39]). An abiotic factor such as the volume of the rearing environment and the presence of a litter therefore plays a role in the development of the body of female spiders.

## 5. Conclusions

My results show that, as for other animal species, abiotic and biotic living conditions influence the locomotor and exploratory behaviours activities of the wandering spider *P. saltans*. The rearing conditions I used influenced positively their foraging behaviour (weight gain) but may have influenced profoundly and negatively the behaviour of adults. My study also highlights the fact that their environment impacts males and females differently. Thus, the modifications of female spiders’ behaviour can be attenuated by physical enrichment of their environment. In contrast, males were more impacted by my laboratory conditions, and simply adding substrate was not sufficient to reduce this impact. These negative changes of *P. saltans*’ behaviour occurred rapidly (in less than 15 days). These results underscore the importance of taking into account the length of time individuals have been kept in the laboratory before drawing conclusions behavioural studies concerning animal well-being.

## Figures and Tables

**Table 1:** Parameters measured for evaluation of locomotor, exploratory and spontaneous activities of a spider in its terrarium and during behavioural tests in a novel environment (open-field arena and radial-arm maze).

Behavioural Events	Parameters Measured
Locomotor activity (Surface explored in cm/min)	Time of mobility Trajectory
Spontaneous activity without displacement (Frequency of behaviour)	“Inactivity” (total immobility) “Abdominal” (abdominal vibrations) “Grooming” (passing a leg or a pedipalp between their chelicereae) “Leg-waving” (raising and lowering a leg of their first pair) “Standing” (standing on hind legs vertically to a wall) “Drumming” (drumming the ground with their first pair of legs)
Exploratory activity Distance covered (cm/min)	Time of mobility Number of arms visited

## Data Availability

The data presented in this study are available on request from the corresponding author.
